# Limnological regime shifts caused by climate warming and Lesser Snow Goose population expansion in the western Hudson Bay Lowlands (Manitoba, Canada)

**DOI:** 10.1002/ece3.1354

**Published:** 2015-01-30

**Authors:** Lauren A MacDonald, Nicole Farquharson, Gillian Merritt, Sam Fooks, Andrew S Medeiros, Roland I Hall, Brent B Wolfe, Merrin L Macrae, Jon N Sweetman

**Affiliations:** 1 Department of Biology, University of WaterlooWaterloo, Ontario, N2L 3G1, Canada; 2 Department of Geography and Environmental Studies, Wilfrid Laurier UniversityWaterloo, Ontario, N2L 3C5, Canada; 3 Department of Geography, York UniversityToronto, Ontario, M3J 1P3, Canada; 4 Department of Geography and Environmental Management, University of WaterlooWaterloo, Ontario, N2L 3G1, Canada; 5 Parks Canada, Western and Northern Service CentreWinnipeg, Manitoba, R3B 0R9, Canada

**Keywords:** Carbon isotopes, climate warming, diatoms, Hudson Bay Lowlands, Lesser Snow Goose, limnology, nitrogen isotopes, paleolimnology, pigments, tundra pond

## Abstract

Shallow lakes are dominant features in subarctic and Arctic landscapes and are responsive to multiple stressors, which can lead to rapid changes in limnological regimes with consequences for aquatic resources. We address this theme in the coastal tundra region of Wapusk National Park, western Hudson Bay Lowlands (Canada), where climate has warmed during the past century and the Lesser Snow Goose (LSG; *Chen caerulescens caerulescens*) population has grown rapidly during the past ∽40 years. Integration of limnological and paleolimnological analyses documents profound responses of productivity, nutrient cycling, and aquatic habitat to warming at three ponds (“WAP 12”, “WAP 20”, and “WAP 21″), and to LSG disturbance at the two ponds located in an active nesting area (WAP 20, WAP 21). Based on multiparameter analysis of ^210^Pb-dated sediment records from all three ponds, a regime shift occurred between 1875 and 1900 CE marked by a transition from low productivity, turbid, and nutrient-poor conditions of the Little Ice Age to conditions of higher productivity, lower nitrogen availability, and the development of benthic biofilm habitat as a result of climate warming. Beginning in the mid-1970s, sediment records from WAP 20 and WAP 21 reveal a second regime shift characterized by accelerated productivity and increased nitrogen availability. Coupled with 3 years of limnological data, results suggest that increased productivity at WAP 20 and WAP 21 led to atmospheric CO_2_ invasion to meet algal photosynthetic demand. This limnological regime shift is attributed to an increase in the supply of catchment-derived nutrients from the arrival of LSG and their subsequent disturbance to the landscape. Collectively, findings discriminate the consequences of warming and LSG disturbance on tundra ponds from which we identify a suite of sensitive limnological and paleolimnological measures that can be utilized to inform aquatic ecosystem monitoring.

## Introduction

Shallow lakes and ponds are abundant in subarctic and Arctic regions and provide important habitat and resources for a variety of wildlife and human populations. Due to their small water volumes and high surface area to volume ratios, they are particularly responsive to multiple environmental stressors, which may lead to limnological regime shifts or rapid transition to a new suite of limnological conditions. Climate warming and changes in avian populations have the potential to influence catchment vegetation, nutrient supply, aquatic productivity, and habitat (e.g., Van Geesr et al. 2007; Côté et al. 2010; Luoto et al. 2014; MacDonald et al. 2014). For example, warming has caused reorganization of diatom communities in Arctic lakes by promoting a shift in dominance from benthic species to epiphytic or planktonic species as recorded in sediment cores (e.g., Douglas et al. 1994; Sovari et al. 2002; Rühland et al. 2003, 2013, 2014; Smol et al. 2005). The influence of avian populations has also been identified as a driver of regime shifts in subarctic and Arctic lakes and ponds (e.g., Luoto et al. 2014), and they have long been recognized to cause disturbance in other habitats (e.g., terrestrial landscapes, salt marshes; Jefferies et al. 2006; Kotanen and Abraham 2013). Notably, in subarctic and Arctic regions experiencing the dual effects of climate warming and changes in avian populations, few studies have comprehensively examined and discriminated limnological responses to these stressors, yet this knowledge is required to assess the status of aquatic resources and to anticipate how they may evolve in the future.

**Figure 1:**
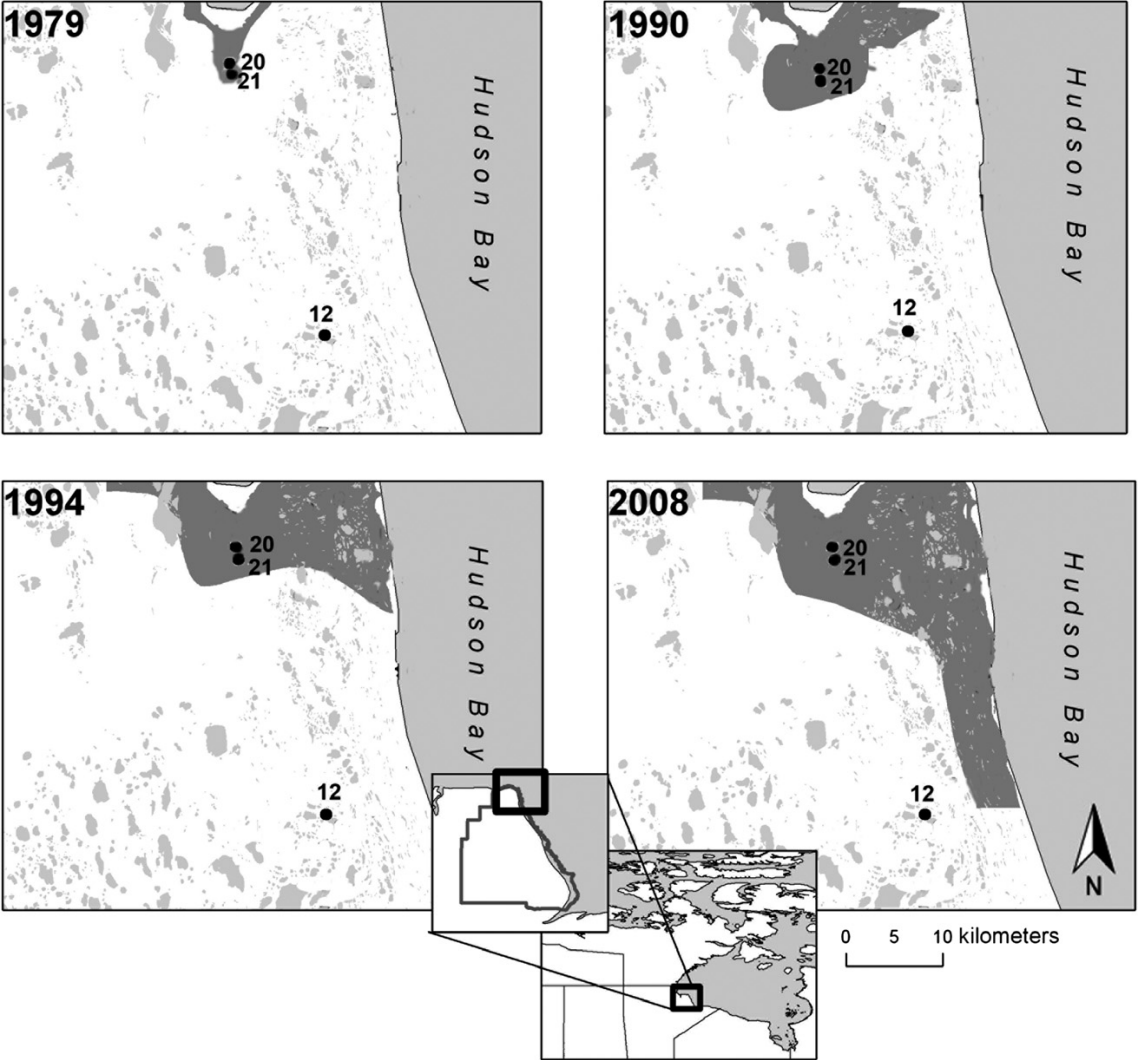
Location of Wapusk National Park (Manitoba, Canada) and the three study ponds (WAP 20, WAP 21, and WAP 12). WAP 20 and WAP 21 are situated in an area of high disturbance by LSG since ∽1979, whereas WAP 12 is located outside of this area as of 2008. Gray regions depict the geographic limits of the LSG distribution at four time periods (based on data provided by Parks Canada in 2010).

**Figure 2:**
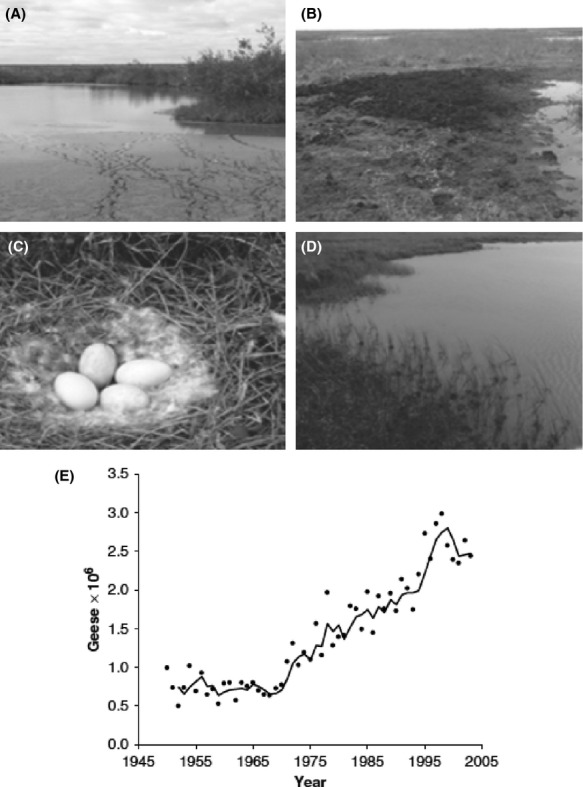
Photographs (A), (B), and (C) depict LSG disturbance in and adjacent to ponds WAP 20 and WAP 21. Photograph (D) is from WAP 12 and depicts a pond with low disturbance from the LSG population. Graph in panel (E) is an estimate of LSG population rise (Abraham et al. [Bibr b1][Bibr b2]; pg. 843). The solid line is a 3-year running average.

Wapusk National Park (WNP; Manitoba, Canada; Fig.1), western Hudson Bay Lowlands, offers opportunity to examine potential limnological regime shifts in response to changes in climate and avian populations in a subarctic setting. Shallow lakes and ponds cover ∽25 to 40% of the surface area, which provide important habitat for wildlife (Parks Canada 2008). The climate of the Churchill region has warmed during the past century, and climate models predict that mean annual temperatures will increase by 3.1°C by 2070 (Macrae et al. 2014). Since the ∽1970s, a rapid increase in the Lesser Snow Goose (LSG; *Chen caerulescens caerulescens*) population (geometric increase of 5–7% per year; Batt et al. 1997; Jefferies et al. 2006) and spatial extent of breeding grounds in the coastal region of WNP has disturbed the landscape and caused substantial changes in vegetation and habitat in the tidal flats (Figs.1 and 2; e.g., Lacobelli and Jefferies 1991; Srivastava and Jefferies 1996; Handa et al. 2002; Jefferies and Rockwell 2002; Jefferies et al. 2006). With the expansion in breeding grounds, the LSG have moved farther inland, and their activities (grubbing and the removal of grasses, construction and occupation of nests, and deposits of feces) are now evident in catchments of many ponds within the coastal fen ecotype of WNP. Parks Canada (2008) has recognized that climate change and the expanding LSG population are potentially altering the ecological integrity of lakes and ponds within WNP. While prior limnological studies in WNP and in the Churchill area have focused on seasonal and short-term investigations (Macrae et al. 2004; Bos and Pellatt, 2012; Eichel et al. 2014; MacDonald et al. 2014; White et al. 2014), sediments in these ponds contain a rich source of paleoenvironmental information (Wolfe et al. 2011; Bouchard et al. 2013) that has yet to be fully exploited to provide a temporal perspective of shifting limnological conditions.

Here, we integrate paleolimnological analyses, which provide insight into ∽250 years of limnological evolution, with three years of water chemistry measurements, to disentangle the limnological responses and identify regime shifts associated with climate warming and LSG population expansion in WNP. We compare three ponds located within the coastal fen ecotype including “WAP 20” and “WAP 21”, whose catchments are strongly disturbed by LSG based on recent field observations, and “WAP 12”, which has no visual evidence of recent LSG disturbance. Findings are used to elucidate the influence of warming and increasing LSG population over time through a suite of limnological and paleolimnological indicators that can be used by agencies to monitor the ongoing consequences of these stressors on aquatic ecosystems.

## Materials and Methods

### Study area

The coastal fen ecotype covers ∽11% of WNP (Parks Canada 2000). Here, the terrestrial vegetation consists mainly of graminoids, sedges (*Carex aquatilis*), and rushes (*Scorpidium scorpoides* and *Scirpus caespitosus*). The study ponds WAP 20 and WAP 21 are situated in catchments located within an area of the coastal fen ecotype subject to the longest observed occupation by LSG (since ∽1979; Fig.1). Within their catchments, we observed abundant deposits of feces, nests, geese eggs, substantial grubbing, and vegetation loss during sampling campaigns in 2010–2012 (Fig.2). In contrast, WAP 12, also located in the coastal fen ecotype, had little to no visual evidence of LSG disturbance and is positioned outside of the disturbed area identified in 2008 maps developed by Parks Canada (Fig.1). WAP 20 and WAP 21 are within 1 km of one another, and WAP 12 is located ∽29 km from WAP 20 and WAP 21. As all three ponds are located within the same geographic region, we assume they are subject to similar climatic conditions. All three ponds are small (<0.30 km^2^) and shallow (<0.5 m deep) and are situated in low-relief terrain consisting of sand to stony silt marine/glaciomarine deposits from the former Tyrrell Sea overlain by muskeg (Dredge and Nixon 1986).

### Limnology

Surface water was collected from the ponds during the spring (early-June), summer (late July), and fall (mid-September) during 2010–2012. Average values for each sampling period (June, July, and September) are reported here. All samples were transported to the Churchill Northern Studies Centre (CNSC) via helicopter for on-site processing. In situ measurements for pH and conductivity were taken at approximately 10- to 15-cm water depth using a YSI 600QS multiparameter probe. Surface water samples collected at each site were passed through an 80-*μ*m mesh to remove large particles and then stored in the dark at 4°C until they were subsampled for subsequent analyses. Samples were measured for a suite of limnological analyses. Here, we report selected variables based on the findings of MacDonald et al. (2014). These include concentration of total Kjeldahl nitrogen (TKN) measured at the Biogeochemistry Lab, University of Waterloo, following standard methods (TKN = Bran Luebbe, Method No. G-189-097; Seal Analytical, Seattle). Carbon isotope composition of dissolved inorganic carbon (*δ*^13^C_DIC_) was measured on water samples collected using 125-ml glass serum bottles with rubber stoppers and syringe needles to expel excess air at field sites. These samples were stored at 4 °C until analysis at the University of Waterloo – Environmental Isotope Laboratory (UW-EIL). Samples for the measurement of C isotope composition of particulate organic matter (*δ*^13^C_POM_) were collected using a phytoplankton net (mesh size = 25 *μ*m) and multiple horizontal tows. After collection, samples were passed through a 63-*μ*m mesh net to remove zooplankton and other large particles, filtered onto pre-ashed Whatman® (GE Healthcare UK Limited, Little Chalfront, UK) quartz filters (0.45-*μ*m pore size), and dried at 60 °C for 24 h in an oven. 12N HCl fumes were then used to remove carbonates from the filters following methods of Lorrain et al. (2003). The acidified filters were analyzed for *δ*^13^C_POM_ at the UW-EIL. Stable C isotope ratios are reported as *δ*^13^C (‰) relative to the Vienna-PeeDee Belemnite (VPDB) standard. The C isotope fractionation factor was estimated from the difference between *δ*^13^C_DIC_ and *δ*^13^C_POM_ (i.e., Δ^13^C_DIC-POM_; Fry 2006).

### Collection of lake sediment cores

Sediment cores were retrieved from the center of the study ponds using a hand-driven rod attached to an open-barrel corer (Glew et al. 2001) in late July 2010 from WAP 20 (core 2: 13.0-cm long, core 3: 15.0-cm long and core 5: 14.5-cm long) and WAP 21 (33.5-cm long), and in September 2011 from WAP 12 (core 1: 25-cm long; core 2: 22.5-cm long). All cores were transported to the CNSC via helicopter, sectioned into 0.5-cm intervals using a vertical extruder (Glew 1988), and stored at 4°C in the dark during shipping and until analysis. Table1 indicates the sampling strategy employed to ensure there was sufficient material for analysis of all parameters for each pond.

**Table 1:** Analytical strategy for the sediment cores

Analysis	WAP 20	WAP 21	WAP 12
Loss on ignition	Core 2, core 3, and core 5	Core 1	Core 1 and core 2
	Every 0.5-cm interval	Every 0.5-cm interval	Every 0.5-cm interval
Chronology	Core 3	Core 1	Core 2
	Every fourth 0.5-cm interval	Every second 0.5-cm interval	Every second 0.5-cm interval
Geochemistry	Core 3	Core 1	Core 1
	Every second 0.5-cm interval	0–19 cm: every second 0.5-cm interval	Every 0.5-cm interval
			19–bottom: every 0.5-cm interval
Diatoms	Core 3	Core 1	Core 1
	Every second 0.5-cm interval	Every second 0.5-cm interval	Every second 0.5-cm interval
Pigments	Core 2	Core 1	Core 1
	Every second 0.5-cm interval	Every second 0.5-cm interval	Every second 0.5-cm interval

### Loss on sequential heating

Subsamples of ∽0.3 ± 0.05 g of wet sediment were analyzed to determine water content, organic matter content, and carbonate content by conducting weight loss on heating (90°C for 24 h), loss on ignition (LOI; 550°C for 1 h), and loss on combustion (LOC; 950°C for 1 h), respectively, following standard methods (Heiri et al. 2001). Mineral matter content was also determined using the residue after LOC.

### Sediment core chronology

To develop the sediment core chronologies, subsamples of dried sediment were analyzed for at least 2 days for activities of ^210^Pb, ^226^Ra (as ^214^Bi and ^214^Pb), and ^137^Cs using an Ortec co-axial HPGe Digital Gamma Ray Spectrometer (#GWL-120-15; Ortec, Oak Ridge, TN, U.S.A.) interfaced with Maestro 32 software (version 5.32, Ortec Oak Ridge, TN, U.S.A.) at the University of Waterloo. Environmental Change Research (WATER) Laboratory. Polypropylene tubes (8 mL; Sarstedt, Numbrecht, Germany; product No. 55.524) were used to pack measured masses of freeze-dried sediment, which were then allowed to equilibrate for at least 2 weeks before analysis. Chronologies were developed using the Constant Rate of Supply (CRS) model (Appleby 2001). The mean activity of ^214^Bi and ^214^Pb was used to estimate background (supported) ^210^Pb activity. Activity of ^137^Cs, which often forms a distinctive peak corresponding to maximum atmospheric fallout from above-ground nuclear weapons testing in 1963 (Appleby 2001), was used as an independent time marker.

### Geochemical analysis of sediment cores

Subsamples were prepared for geochemical analysis following standard methods described by Wolfe et al. (2001). Subsamples of wet sediment were treated with 10% HCl to remove carbonates and subsequently rinsed repeatedly with de-ionized water until a neutral pH was reached. The samples were then freeze-dried, and the fine fraction (<500 *μ*m) was analyzed for organic C and N elemental and isotope composition at the UW-EIL. The C-to-N ratio was calculated using percent dry weight of organic C and N. Stable C and N isotope ratios are reported as *δ*^13^C_org_ (‰) units relative to the Vienna-Peedee Belemnite (VPDB) standard and *δ*^15^N (‰) units relative to atmospheric N (AIR), respectively. For WAP 20, average reproducibility was ±1.91% and ±0.63% for elemental organic C and N, respectively, and ±0.16 ‰ and ±0.10 ‰ for *δ*^13^C_org_ and *δ*^15^N, respectively. For WAP 21, average reproducibility was ±0.54% and ±0.04% for elemental organic C and N, respectively, and ±0.27 ‰ and ±0.08 ‰ for *δ*^13^C_org_ and *δ*^15^N, respectively. For WAP 12, average reproducibility was ±1.92% and ±0.13% for elemental organic C and N content, respectively, and ±0.01 ‰ and ±0.02 ‰ for *δ*^13^C_org_ and *δ*^15^N, respectively.

### Sedimentary diatom analysis

For analysis of diatoms, subsamples (∽0.3 ± 0.05 g) of wet sediment were treated with 10% HCl for 24 h to remove carbonates. After allowing sufficient time for diatoms to settle to the bottom of the sample tubes, the supernatant was siphoned off and de-ionized water was added to dilute the acid. This sequence was then repeated several times until a neutral pH was reached. Subsamples were then digested with strong acids (50:50 concentrated sulfuric:nitric acids by volume) to remove organic materials. After allowing sufficient time for diatoms to settle to the bottom of the sample tubes, the supernatant was siphoned off and samples were rinsed with de-ionized water. This sequence was repeated until a neutral pH was obtained. The resulting cleaned diatom slurries were then dispensed onto circular coverslips and allowed to dry before mounting them onto microscope slides using Naphrax mounting medium. For each sample, approximately 300 diatom valves were identified to the lowest possible taxonomic level at 1000× magnification using a Zeiss Axioskop II Plus (Carl Zeiss, Jena, Germany) compound light microscope fitted with differential interference contrast optics. Diatom identifications were based on the keys of Krammer and Lange-Bertalot (1986–1991) and Lavoie et al. (2008).

### Sedimentary pigment analysis

Standard methods (Reuss et al. 2010) were used to prepare, extract, and analyze pigment concentrations from subsamples (∽0.3 ± 0.05 g) of wet sediment at the University of Waterloo WATER Lab. A WATERS 2695 HPLC was used with the reverse-phase high-performance liquid chromatography method by Mantoura and Llewellyn (1983) and modified by Leavitt et al. (1989). Pigments were identified based on their retention times and elution sequence, and comparison of their spectra compared to known standards (Jeffrey et al. 1997). Pigment concentrations are expressed as nanomoles per gram of organic matter.

### Stratigraphic analysis

To assist identifying the timing of regime shifts in the study ponds, we determined breakpoints in stratigraphic profiles individually for each of the physical and geochemical variables via use of generalized linear models with piecewise linear relationships having a fixed number of breakpoints. The average fitted breakpoint date and relevant standard error for all physical and geochemical variables was calculated, which we present in stratigraphic plots and the text below. The average fitted breakpoint date and relevant standard error of the physical and geochemical variables calculated for each pond were also applied to the biological data. Numerical procedures were conducted with the use of R statistical language v3.1.1 with the rioja and segmented libraries.

## Results and Interpretation

### Sediment core chronologies

**Figure 3:**
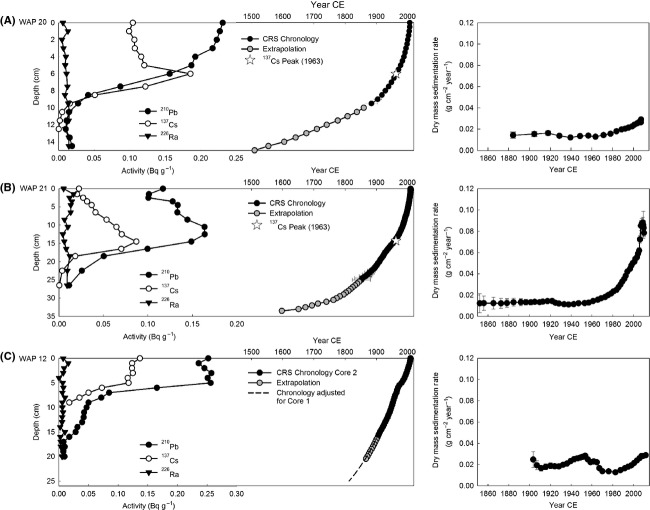
Left panels present scatterplots showing activity profiles for the radioisotopes and depth–age profiles for (A) WAP 20, (B) WAP 21, and (C) WAP 12 sediment cores. Right panels present sedimentation rates. Error bars represent standard deviations.

Total ^210^Pb activity declined steadily with depth in the sediment core from WAP 20, from 0.231 Bq g^−1^ at the top to 0.010 Bq g^−1^ at 12.5 cm (Fig.3A). The mean activity of ^226^Ra (0.011 Bq g^−1^) was used to estimate supported ^210^Pb. Total ^210^Pb activity was almost equivalent to the estimated supported ^210^Pb at 12.5 cm (0.010 Bq g^−1^), indicating that background was likely reached at this depth. Using the CRS model and linear extrapolation, a basal date of 1467 was determined for the sediment core from WAP 20. Peak concentration in ^137^Cs (0.186 Bq g^−1^) at 6 cm occurred at the CRS date of 1963, which corresponds to the expected year of peak fallout. The sedimentation rate fluctuated between 0.013 g cm^−2^ year^−1^ and 0.016 g cm^−2^ year^−1^ before 1977 and then increased to a maximum of 0.029 g cm^−2^ year^−1^ at the top of the core.

In contrast to WAP 20, total ^210^Pb activity did not decline steadily down core at WAP 21 (Fig.3B). Instead, peak activity (0.164 Bq g^−1^) occurred at 12.5 cm and declined at depths above and below. Total ^210^Pb activity declined to 0.011 Bq g^−1^ at 26.5 cm depth and was near equivalent to the estimated supported ^210^Pb at 26.5 cm (0.011 Bq g^−1^), indicating that background was reached at this depth. A basal date of 1597 was determined for the core. Peak concentration of ^137^Cs (0.087 Bq g^−1^) occurred at 14.5 cm and corresponded to a CRS date of 1957, which is close to the expected date of 1963. The sedimentation rate fluctuated between 0.011 g cm^−2^ year^−1^ and 0.014 g cm^−2^ year^−1^, before it began a steady increase during 1961–1978 to 0.021 g cm^−2^ year^−1^. The sedimentation rate then increased exponentially and reached a maximum value of 0.088 g cm^−2^ year^−1^ at the top of the core.

Total ^210^Pb activity was relatively constant (∽0.25 Bq g^−1^) in the upper 5 cm of the core from WAP 12 (Fig.3C). Between 5 cm and 17 cm, ^210^Pb activity declined to 0.008 Bq g^−1^. Total ^210^Pb activity at 17 cm was close to the estimated supported ^210^Pb (0.006 Bq g^−1^), indicating that background was likely reached at this depth. Using the CRS model, the base of unsupported ^210^Pb in WAP 12 core 2 was estimated to be 1904. Extrapolation of the age–depth relation to core 1 gave a basal date of 1813. ^137^Cs activity did not display a clear peak in the core, likely due to the mobile nature of Cs in the highly organic sediments. Sedimentation rates fluctuated between 0.013 g cm^−2^ year^−1^ and 0.029 g cm^−2^ year^−1^ throughout the record. Peak sedimentation rates occurred in 1953 and at the top of the core (0.028 and 0.029 g cm^−2^ year^−1^, respectively). Although sedimentation rate has increased during the past few decades, values remain within the historical range. Near-constant ^210^Pb activities at the top of the sediment core from WAP 12 are similar to ^210^Pb profiles in other pond sediment cores in the Churchill region (Wolfe et al. 2011) and are likely due to increase in sedimentation rate, because sediment mixing is unlikely based on fluctuating values of many parameters during this interval (see below and Bouchard et al. 2013).

### Limnological evolution of the ponds

Stratigraphic profiles for organic matter content (and organic matter flux for WAP 21), mineral matter content, calcium carbonate content, organic C content, N content, C/N, organic C isotope composition, and N isotope composition show prominent temporal variations (Figs6). Stratigraphic profiles shown also include the percent abundance of *Fragilaria pinnata* (Ehrenberg) and *Denticula kuetzingii* (Kützing), which were the two most dominant diatom taxa in sediment cores from all three ponds, and concentrations of chlorophyll *a* to represent total algal abundance and concentrations of aphanizophyll or canthaxanthin to represent abundance of potentially nitrogen-fixing cyanobacteria (Figs6). This broad array of paleolimnological measurements allows us to efficiently characterize past limnological conditions and intervals and identify changes during the past 200–250 years.

**Figure 4:**
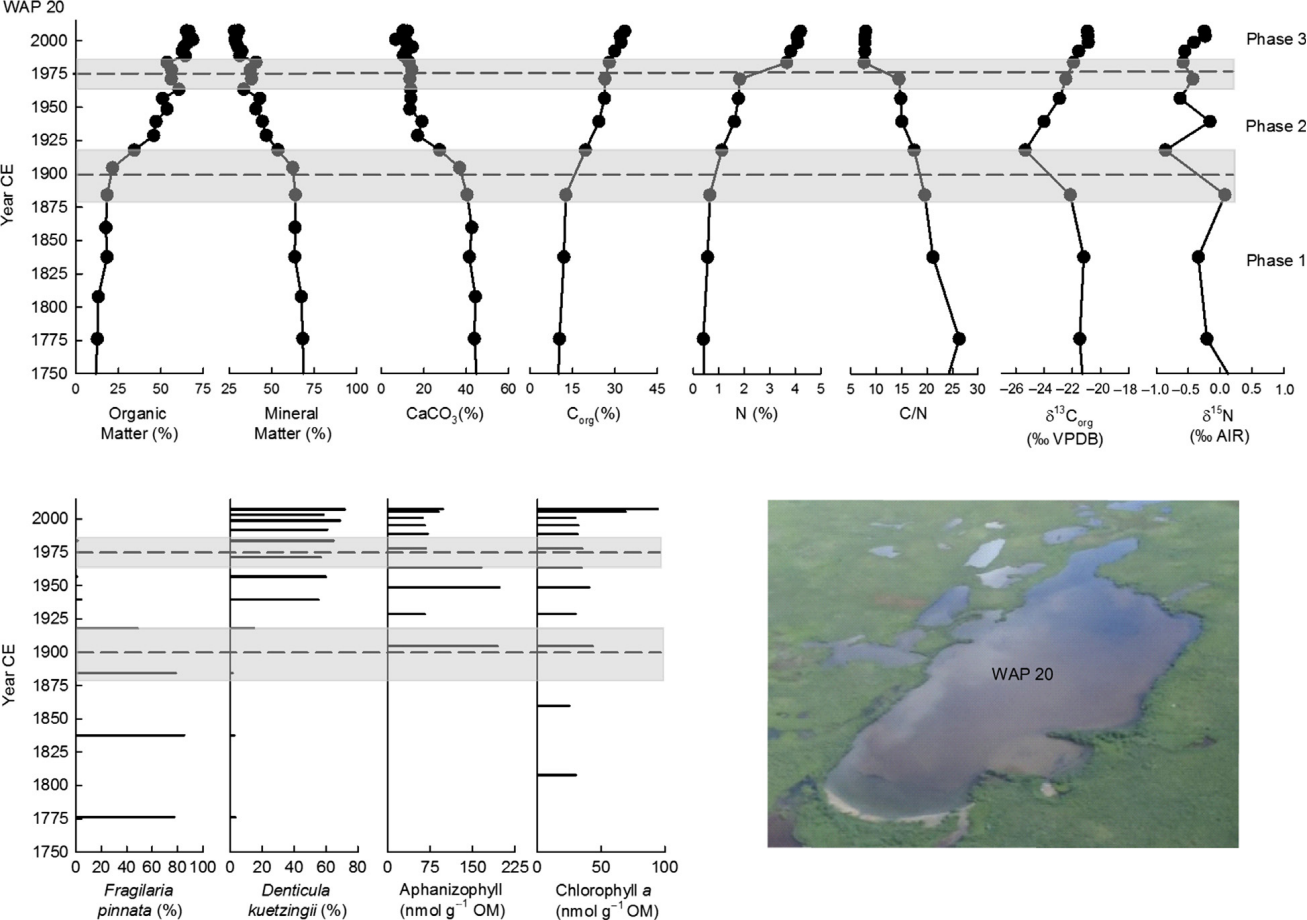
Stratigraphic profiles of selected paleolimnological variables for WAP 20. The vertical axis presents the age of the sediment core, as estimated from the ^210^Pb analysis. The average fitted breakpoint date of physical and geochemical variables is shown by a dashed line with the relative error indicated with a gray bar. The breakpoints were also applied to diatom and pigment profiles.

**Figure 5:**
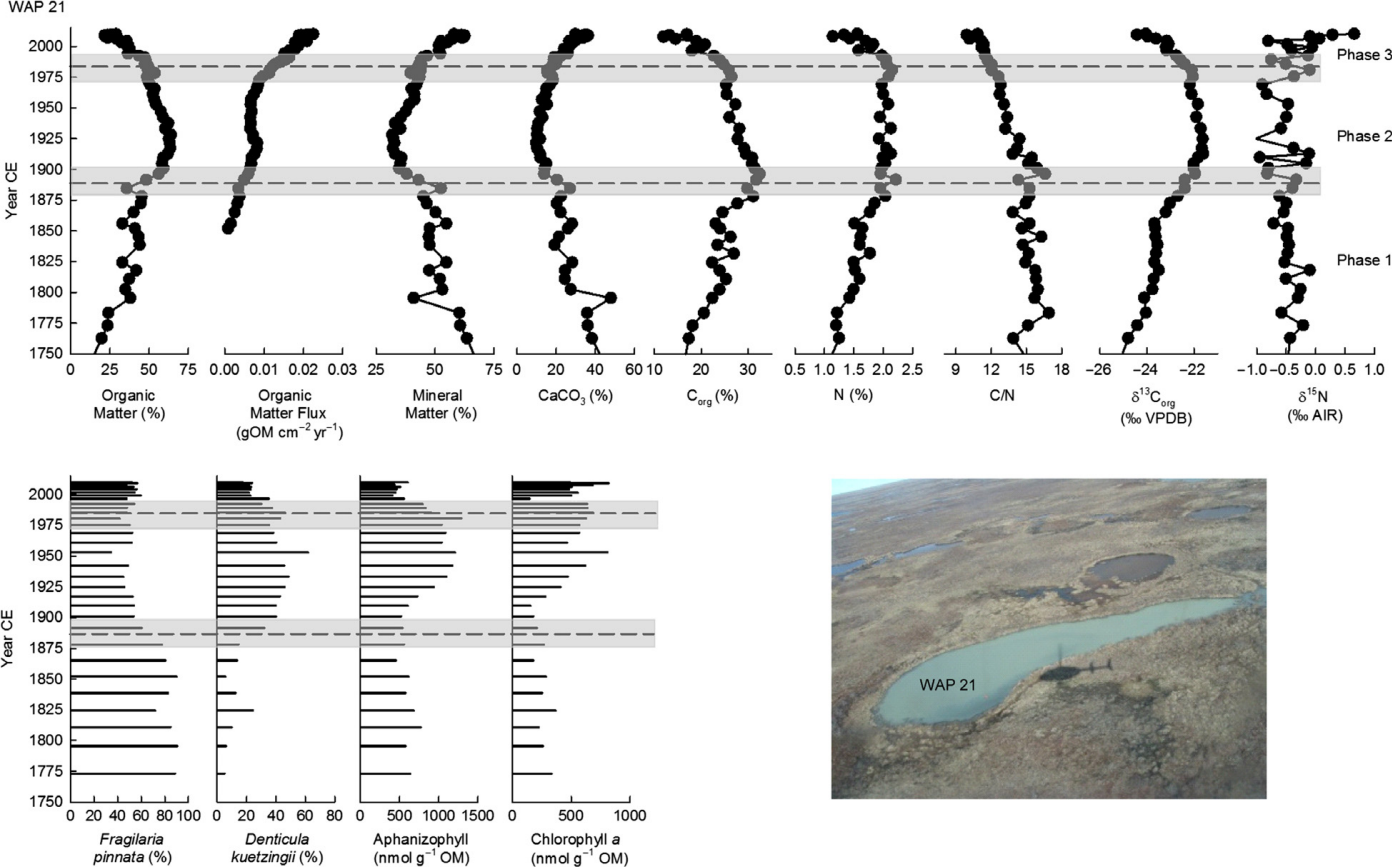
Stratigraphic profiles of selected paleolimnological variables for WAP 21. The vertical axis presents the age of the sediment core, as estimated from the ^210^Pb analysis. The average fitted breakpoint date of physical and geochemical variables is shown by a dashed line with the relative error indicated with a gray bar. The breakpoints were also applied to diatom and pigment profiles.

**Figure 6:**
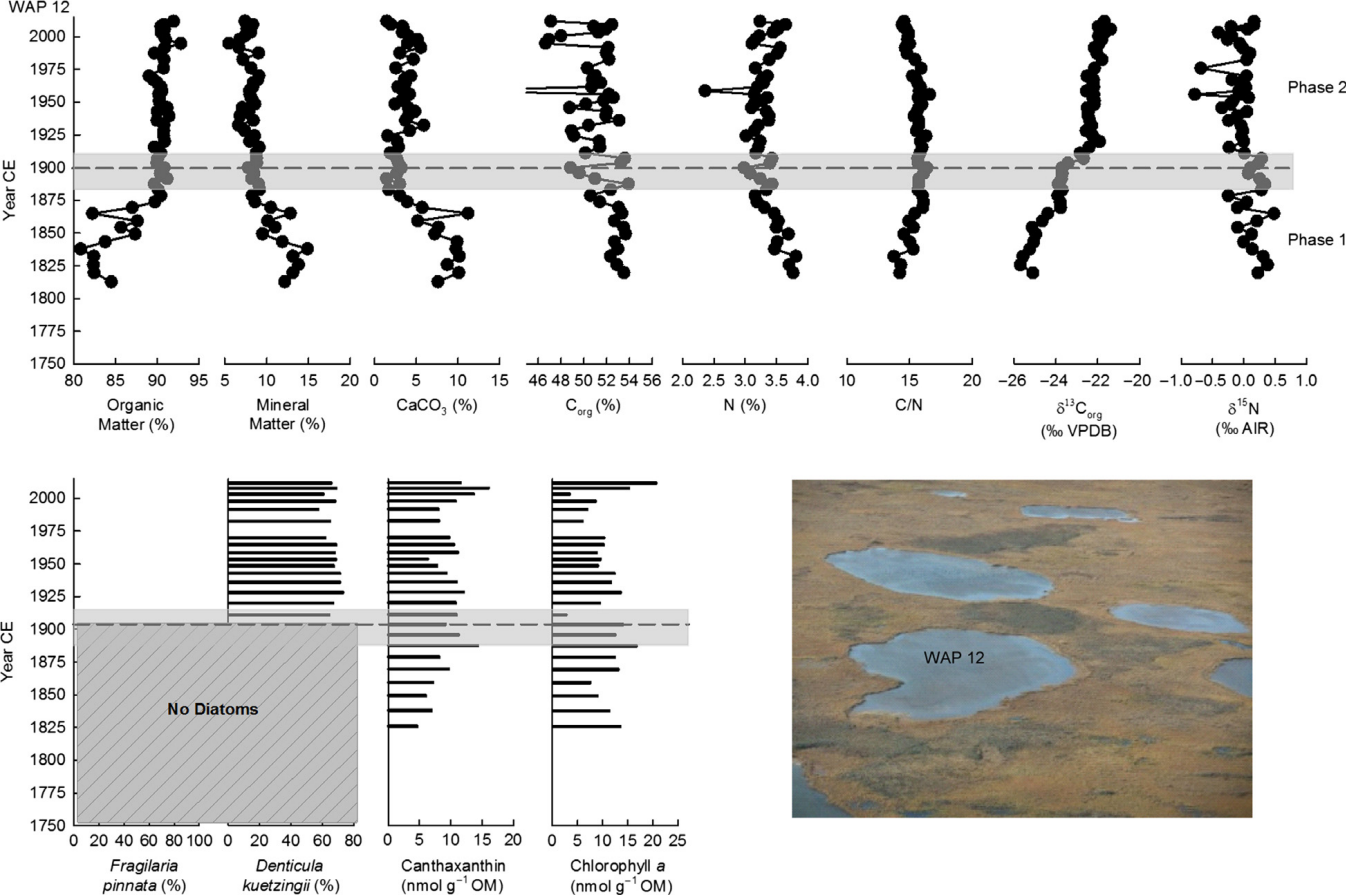
Stratigraphic profiles of selected paleolimnological variables for WAP 12. The vertical axis presents the age of the sediment core, as estimated from the ^210^Pb analysis. The average fitted breakpoint date of physical and geochemical variables is shown by a dashed line with the relative error indicated with a gray bar. The breakpoints were also applied to diatom and pigment profiles.

Minimal changes occurred in the older strata of all three sediment records, and to provide sufficient temporal context to evaluate the 20th century changes, only results post-1750 (post-1813 for WAP 12) are reported here. Breakpoint analyses on the physical and geochemical variables were used to guide our identification of the timing of major limnological changes. Breakpoint analyses indicated significant changes at ∽1900 (±49 years) and ∽1975 (±15 years) for WAP 20, ∽1893 (±22 years) and ∽1983 (±25 years) for WAP 21, and ∽1900 (±31 years) for WAP 12. Based on these breakpoints, three phases of different limnological conditions were identified at WAP 20 and WAP 21 and two phases at WAP 12. The phase boundaries coincide with marked changes in the composition of diatom communities and abundance of algal pigments.

#### Phase 1 (pre-1750 to ∽1893–1900)

During the first phase of the sediment records (pre-1750 to ∽1893–1900), values of the physical, geochemical, and biological variables remained relatively constant or displayed gradual trends over time. Despite relatively coarse temporal resolution, subtle trends are apparent in the core from WAP 20 (Fig.4). Organic matter content increased gradually (∽11 to 18%) throughout this phase, while mineral matter (∽69 to 63%) and calcium carbonate content (∽45 to 40%) decreased. Organic C content remained around 11%, and N content remained around 0.5%. Values of the C/N ratio decreased gradually during this interval (26 to 20), but were relatively high overall. Values of *δ*^13^C_org_ (∽−21 ‰) and *δ*^15^N (−0.25 ‰) were relatively constant. Diatom community composition and pigment concentrations varied only slightly. Diatom assemblages were dominated by *Fragilaria pinnata* (∽80%), and sediments contained relatively low concentrations of chlorophyll *a* (∽25 to 30 nmol g^−1 ^OM).

During Phase 1 in the WAP 21 sediment core, gradual changes are evident in several of the parameters (Fig.5). Organic matter content increased gradually but substantially (11 to 48%), and mineral matter (69 to 43%) and carbonate content (45 to 20%) decreased correspondingly. Organic C content increased (16 to 32%), and N content increased from ∽1 to 2%. Values of the C/N ratio were relatively constant and high (15–16). Values of *δ*^13^C_org_ increased gradually (−25 to −22 ‰), and *δ*^15^N fluctuated around −0.5 ‰. Diatom community composition and pigment concentrations varied only slightly. Diatom assemblages were dominated by *Fragilaria pinnata* (80%), and sediments contained relatively moderate concentrations of the pigments aphanizophyll (∽500 to 700 nmol g^−1^ OM) and relatively low concentrations of chlorophyll *a* (∽200 to 300 nmol g^−1^ OM).

In the core from WAP 12, gradual changes are evident from the base of the core (∽1813) until ∽1900 (Fig.6). Organic matter increased (84 to 90%), while mineral matter (12 to 9%) and calcium carbonate content (8 to 3%) declined. Organic C and N content fluctuated around ∽53% and 3.5%, respectively, and C/N ratios were relatively high (14 to 16). Values of *δ*^13^C_org_ increased slightly (−25 to −24 ‰), and *δ*^15^N remained near 0 ‰. Diatoms were not in sufficient abundance or not sufficiently preserved in sediments deposited during this phase to allow enumeration, and concentrations of pigments canthaxanthin and chlorophyll *a* fluctuated slightly but were relatively low (∽4–11 nmol g^−1^ OM and ∽9–16 nmol g^−1^ OM, respectively).

Overall, results indicate that limnological conditions at WAP 20, WAP 21, and WAP 12 gradually transitioned toward increased aquatic productivity during Phase 1, as indicated by gradual increases in organic matter content, organic C content, and *δ*^13^C_org_. Increasing algal demand on DIC usually leads to ^13^C enrichment of DIC and phytoplankton, which is recorded in pond sediments (Meyers and Teranes 2001; Meyers 2003). High C/N ratios in the sediment records from all three ponds suggest that aquatic productivity occurred under N-limited conditions. Likewise, all three sediment cores contained *δ*^15^N values close to 0 ‰, possibly indicative of fixation of atmospheric N (Talbot 2001). At WAP 21 and WAP 12, sediments contained measurable concentrations of the pigments aphanizophyll and canthaxanthin, respectively. These pigments are produced by potentially N-fixing taxa (e.g., Leavitt and Hodgson 2001), a finding that is consistent with N-limited conditions inferred from C/N ratios and *δ*^15^N values. However, concentrations of these pigments and chlorophyll *a* were relatively low during this phase, suggesting low algal productivity. Low productivity in the ponds is further substantiated by the diatom assemblage data that reveal information about conditions of pond habitat. *Fragilaria pinnata* was the dominant taxon in WAP 20 and WAP 21 during this phase, while diatom abundance was too low in sediments from WAP 12 to allow enumeration. *Fragilaria pinnata* is an epipsammic and alkaliphilic diatom taxon that can live on mineral grains and is considered to indicate low light availability caused by minerogenic turbidity, which generates poor habitat for epiphytic and planktonic diatom taxa (Rühland and Smol 2005; Smol et al. 2005). Dominance by *Fragilaria pinnata,* thus, indicates that the epiphytic and plankton habitats did not support considerable growth of algae. Instead, dominance by *Fragilaria pinnata* suggests that most of the algal growth occurred in benthic (and mainly epipsammic) habitat. For WAP 20 and WAP 21, shoreline erosion of inorganic sediment may have provided the input of material for *Fragilaria pinnata* to grow and flourish. In contrast, WAP 12 was likely not experiencing shoreline erosion during this phase as indicated by the higher organic matter content and higher organic C accumulation in the sediment core. Low input of inorganic matter would have limited the benthic habitat available for *Fragilaria pinnata*.

#### Phase 2 (∽1893–1900 to 1975–1983)

Paleolimnological analyses identified marked changes in sediment records from all three ponds around 1893–1900 (Figs6). At WAP 20, sedimentary content of organic matter increased after ∽1900 (∽18 to 56%), and content of mineral matter (∽64 to 38%) and calcium carbonate declined (∽40 to 13% by 1949 and then remained relatively constant). Organic C content (∽13 to 26%), N content (∽0.6 to 2.0%), and *δ*^13^C_org_ (−25 to −22 ‰) also increased at WAP 20. Values of the C/N ratio continued to decline gradually (20–14), but remained relatively high, and *δ*^15^N fluctuated around −0.5 ‰. Composition of diatom assemblages changed abruptly during this phase from dominance by the epipsammic *Fragilaria pinnata* (from ∽80 to ∽1%) to dominance by *Denticula kuetzingii* (1 to 60%), a taxon commonly found in benthic biofilms in ponds near Churchill, Manitoba (Macrae 1998; White et al. 2014). The N-fixing cyanobacterial pigment aphanizophyll first appeared in the sediment record in Phase 2 and reached peak concentrations (∽197 nmol g^−1^ OM). Additionally, concentrations of chlorophyll *a* increased (∽35 nmol g^−1 ^OM).

At WAP 21, organic matter content initially increased (56 to 63%) after ∽1893 until ∽1934 and then decreased (to 49%). However, the organic matter flux continued to increase throughout this phase (0.006 to 0.011 g OM cm^−2^ year^−1^). Mineral matter (37 to 43%) and calcium carbonate (14 to 18%) content increased. Organic C content decreased (32 to 26%), while N content fluctuated around 2%, and C/N values declined slightly (17 to 13) but remained relatively high. *δ*^13^C_org_ values fluctuated around −22 ‰, and *δ*^15^N fluctuated around 0.5 ‰. Similar to WAP 20, the diatom community composition changed abruptly (but earlier; ∽1880) from dominance by *Fragilaria pinnata* to dominance by *Denticula kuetzingii*, which reached peak abundance of 62% at ∽1952. Aphanizophyll content increased to peak concentrations (∽1200 nmol g^−1^ OM) during this phase. Additionally, concentrations of chlorophyll *a* increased to peak concentrations (∽800 nmol g^−1 ^OM).

At WAP 12, changes occurred between ∽1875 and ∽1900. Organic matter content increased to ∽90% and then remained constant to the top of the core. Mineral matter content decreased and then fluctuated around 8%. CaCO_3_ content decreased and then fluctuated around 3% until 1925, when it increased slightly to 5% and fluctuated around this level to the top of the sediment record. Organic C and N content became more variable during this phase and fluctuated mostly between 37–53% and around 3%, respectively. Values of the C/N ratio were relatively constant after ∽1875 and remained at ∽15 to the top of the core. *δ*^13^C_org_ was relatively constant around −24 ‰ from ∽1875 to 1900 and then increased to −22 ‰ and remained relatively stable in the upper sediments. *δ*^15^N showed an initial increase to about 0.3 ‰ and then fluctuated between −0.8 and 0 ‰ until the top of the core. Diatoms became sufficiently abundant to allow enumeration at ∽1911 and were dominated by *Denticula kuetzingii*, similar to WAP 20 and WAP 21. Concentrations of canthaxanthin increased slightly at ∽1875 and fluctuated between 8 and 11 nmol g^−1^ OM until 2003 when an increase to peak concentrations of 16 nmol g^−1^ OM occurred in ∽2008. Chlorophyll *a* showed larger fluctuations in concentrations (average 10 nmol g^−1^ OM) with an increase to 20 nmol g^−1^ OM at the top of the core.

Together, the stratigraphic records from WAP 20, WAP 21, and WAP 12 suggest a marked change began during the late 1800s and early 1900s, characterized by increased aquatic productivity and associated shifts in limnological conditions and available habitat. The increase in organic matter content (or organic matter flux), pigment concentrations, and shift in dominant diatom taxa can be explained by reduced rates of shoreline erosion and input of inorganic material, leading to less turbid conditions, increased light penetration, and higher aquatic productivity. Higher productivity is also supported by increased values of *δ*^13^C_org_. The increase in productivity likely also fostered a high demand for N and continued N limitation based on *δ*^15^N values close to 0 ‰, high C/N values, and increased concentrations of cyanobacterial pigments aphanizophyll and canthaxanthin produced by potentially N-fixing taxa. In addition, the shift in diatom community composition to dominance of *Denticula kuetzingii* indicates a change in available habitat in the ponds from mineral grains to benthic biofilm.

#### Phase 3 (∽1975–1983 to 2010)

Marked stratigraphic changes in the physical and geochemical variables, although not all in the same direction, are apparent in sediment cores from WAP 20 and WAP 21 beginning in the mid-1970s, but they are notably absent in the core from WAP 12 (Figs6). At WAP 20, increases in sedimentation rate (Fig.3) and continued increases in organic matter content (∽56 to 65%) occurred, while mineral matter (∽38 to 30%) and calcium carbonate (∽14 to 10%) content declined. Organic C (∽26 to 33%) and N (2 to 4%) content increased, and the C/N ratio declined substantially (14 to 8). *δ*^13^C_org_ increased initially from −22 to −21 ‰ and then stabilized for the remainder of the core at −20.9 ‰ after 1998. *δ*^15^N values increased (−0.5 to 0.2 ‰). *Denticula kuetzingii* remained the dominant taxon during this phase. Aphanizophyll content declined abruptly to 67 nmol g^−1^ OM and remained relatively constant until the top 1 cm of the core when an increase to 97 nmol g^−1^ OM occurred. Chlorophyll *a* concentrations fluctuated around 35 nmol g^−1^ OM until the top 1 cm of the core when concentrations increased to 94 nmol g^−1^ OM.

At WAP 21, the sedimentation rate increased exponentially after the mid-1970s (Fig.3), which influenced patterns of change in some of the sedimentary variables. Consequently, values of organic matter are more informative when expressed as flux rate than as concentration. During Phase 3, the flux of organic matter increased (0.01 g cm^−2^ year^−1^ to 0.02 g cm^−2^ year^−1^), while C/N ratios declined (12 to 10). Calcium carbonate content increased (∽18–35%), while C_org_ and N content declined to ∽17% and 1.6%, respectively. *δ*^13^C_org_ decreased (−22 to −24 ‰) and *δ*^15^N increased (−0.5 to 0.7 ‰) slightly during this phase. *Denticula kuetzingii* decreased in percent abundance around ∽1990 to ∽17% at the top of the core, while *Fragilaria pinnata* increased to ∽52% at the top of the core. Aphanizophyll concentration declined to 350 nmol g^−1^ OM and then increased in the top sample to ∽604 nmol g^−1^ OM. Chlorophyll *a* concentrations showed no noticeable trend, and values fluctuated between 150 and 817 nmol g^−1^ OM during this phase.

Increasing concentration or flux of organic matter indicates that aquatic productivity continued to increase in WAP 20 and WAP 21 during Phase 3. At WAP 21, the marked increase in calcium carbonate content may be due to calcite precipitation under conditions of high productivity and pH (Wetzel 2001). This likely accounts for dilution of organic matter, C_org,_ and N content, as well as the increase in *Fragilaria pinnata* due to increased inorganic material for this diatom to grow on. The decrease in C/N ratios, increase in *δ*^15^N values, and the decline in concentration of the pigment aphanizophyll for both WAP 20 and WAP 21 indicate that there was an increase in supply of N to these ponds. As stated above, increasing aquatic productivity often corresponds to increasing *δ*^13^C_org_ in lake sediment cores (Meyers and Teranes 2001; Meyers 2003). However, *δ*^13^C_org_ decreases at WAP 21 and increases only slightly at WAP 20 before attaining constant values after ∽1998. Nonetheless, we attribute these patterns to increasing aquatic productivity because such trends can develop when chemically enhanced CO_2_ invasion occurs, which leads to a decline in *δ*^13^C_DIC_ (Herczeg and Fairbanks 1987), as we describe in the following section. Notably, these inferred limnological changes post-1975 are not evident in the sediment record from WAP 12. Additionally, chlorophyll *a* concentrations were generally lowest in WAP 12 and highest in WAP 21, and the difference increased near the top of the core, providing further support for the stimulation of aquatic productivity at WAP 20 and WAP 21 in recent years.

#### Modern limnology

**Figure 7:**
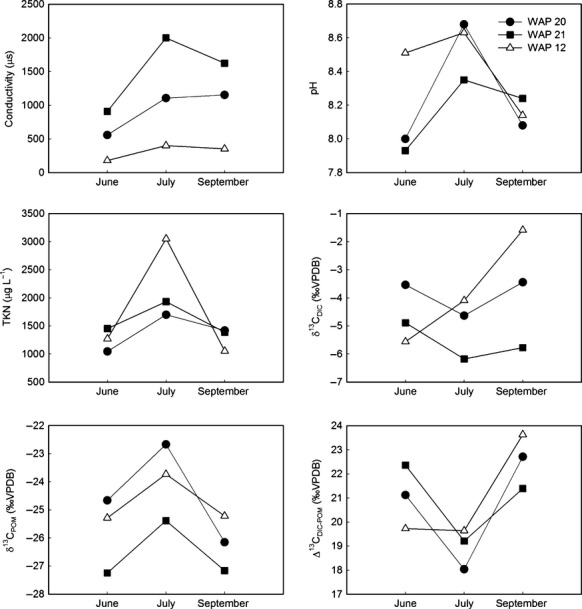
Line plots depicting average seasonal values for selected limnological parameters of ponds WAP 20, WAP 21, and WAP 12 based on findings of MacDonald et al. (2014). Values represent the average of samples collected in early-June 2010–2012, late July 2010–2012, and mid-September 2010–2012. Values for WAP 12 July do not include July 2010 as the pond desiccated, and thus, samples could not be collected.

Average values (2010–2012) of several water chemistry variables distinguish present-day limnological conditions of WAP 20 and WAP 21 from those at WAP 12, particularly in July (Fig.7). Conductivity was much higher at WAP 20 and WAP 21 than at WAP 12. At all three ponds, pH was alkaline, but WAP 20 and WAP 21 had much larger increases in pH between June and July than at WAP 12. All three ponds had lower TKN concentrations in June followed by higher values in July and a return to lower values in September. However, TKN concentrations were substantially lower at WAP 20 and WAP 21 in July than at WAP 12. Values of *δ*^13^C_DIC_ at WAP 20 and WAP 21 decreased between June and July, whereas *δ*^13^C_DIC_ increased throughout the ice-free season at WAP 12. Although seasonal patterns in *δ*^13^C_POM_ were similar among the ponds, C isotope fractionation (Δ^13^C_DIC-POM_) declined sharply at WAP 20 and WAP 21 in July, whereas values at WAP 12 were similar in July and June.

The above limnological features mainly reflect differences in LSG disturbance and associated nutrient behavior at WAP 20 and WAP 21 in comparison with WAP 12. Higher conductivity at WAP 20 and WAP 21 compared to WAP 12 is unlikely to have been caused by differences in evaporative concentration, as WAP 12 generally undergoes greater mid-summer pond-water isotopic enrichment (data not shown). Instead, higher conductivity at WAP 20 and WAP 21 is likely due to higher erosional input of dissolved ions to the ponds than at WAP 12, caused by the removal of catchment vegetation by the LSG population. Lower TKN concentrations at WAP 20 and WAP 21 may be a result of more rapid uptake of nutrients by the benthic biofilm. Experimental nutrient additions by Eichel et al. (2014) demonstrated that the benthic biofilm rapidly assimilates N and P. As discussed in detail by MacDonald et al. (2014), the seasonal increase in *δ*^13^C_DIC_ values at WAP 12 is consistent with preferential use of ^12^C by algae during photosynthesis when C supply exceeds C demand (and which commonly leads to ^13^C-enrichment in the organic fraction of lake sediment profiles under conditions of increasing productivity), whereas the decline in July *δ*^13^C_DIC_ values at WAP 20 and WAP 21 is likely due to chemically enhanced CO_2_ invasion. The latter may occur under conditions of high productivity, high C demand, and high pH, resulting in strong kinetic C isotope fractionation as CO_2_ enters from the atmosphere and decline of *δ*^13^C_DIC_ (Herczeg and Fairbanks 1987; Takahashi et al. 1990; Bade et al. 2004). Evidently, this signal has been recorded by muted and declining trends in *δ*^13^C_org_ in the uppermost stratigraphic intervals of the sediment records from WAP 20 and WAP 21, respectively (Figs4 and 5). Further support for the interpretation of higher C demand at WAP 20 and WAP 21 in July, compared to WAP 12, is the decline in Δ^13^C_DIC-POM_.

## Discussion

**Figure 8:**
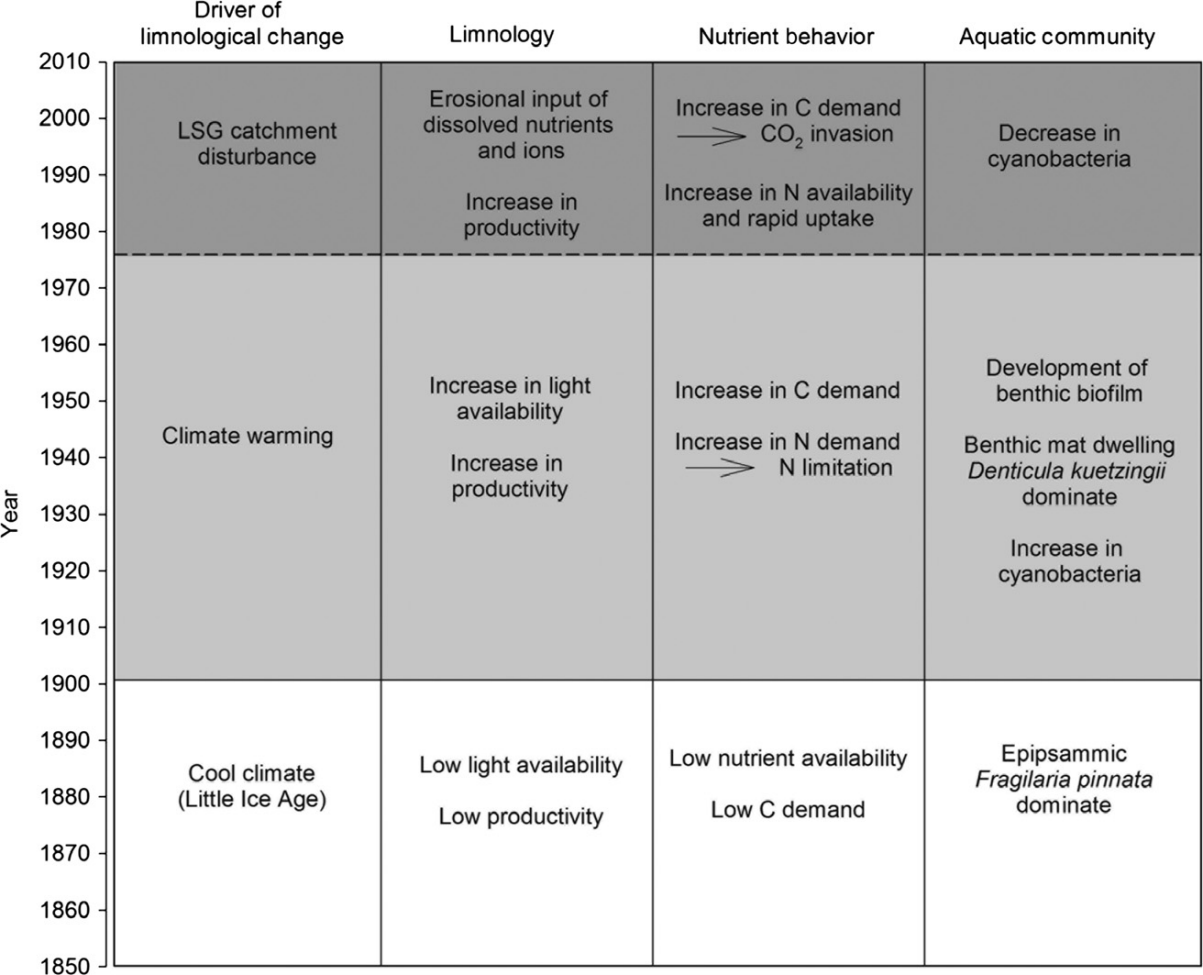
Depiction of the evolution of pond limnology, nutrient behavior, and aquatic community in response to the different drivers of change. The solid horizontal line represents the limnological regime shift that all ponds experienced in response to climate warming, and the dashed horizontal line represents the limnological regime shift that only the LSG-disturbed ponds (WAP 20 and WAP 21) experienced.

Regime shifts in shallow lakes and ponds are rapid changes due to a stressor that causes modifications to the dynamics, organization, and feedbacks of the particular ecosystem resulting in a shift to a different state (e.g., Scheffer and Carpenter 2003; Anderson et al. 2009). These shifts can be characterized by distinct habitats and life forms, and persistence of the new state for a substantial period of time (e.g., Beisner et al. 2003; Scheffer and Carpenter 2003). Limnological regime shifts have been identified in a number of different ecosystems, but their documentation has primarily been based on studies of temperate and boreal lakes and has largely focused on the effects of changing nutrient loads on aquatic vegetation and turbidity (e.g., Scheffer et al. 1993; Bayley and Prather 2003; Scheffer and Carpenter 2003; Bayley et al. 2007). Typically, shallow lakes in temperate and boreal locations switch from a clear-water, macrophyte-dominated state under conditions of low nutrient supply, to a turbid state dominated by phytoplankton as nutrient supply increases and exceeds a threshold (Scheffer et al. 1993). Here, we show that, based on integrating the results from analyses of sediment records and water chemistry, three phases of limnological conditions during the past ∽200–250 years were identified at the ponds located in catchments disturbed by LSG (WAP 20 and WAP 21), whereas only two phases were evident at the nondisturbed pond (WAP 12). As we discuss below, the rapid phase transitions are consistent with aspects of regime shifts that have been documented in shallow temperate and boreal lakes, but we show they occur also in a subarctic setting. Furthermore, our study distinguishes limnological regime shifts driven, not only by changes in available habitat due to warming, but also by the transfer of nutrients from the catchments to the ponds in response to waterfowl disturbance (summarized in Fig.8 and discussed below).

### Limnological regime shift caused by climate warming

Prior to the 20th century (Phase 1), pond conditions can generally be characterized by relatively low nutrient availability, low aquatic productivity, and high mineral matter content of the sediment (with the exception of WAP 12, likely due to lower rates of shoreline erosion). These conditions are consistent with the cold, turbid, low-light conditions of the Little Ice Age (LIA; Overpeck et al. 1997), but abrupt stratigraphic changes occurred in all three ponds during the late ∽1800s to the early ∽1900s (initiation of Phase 2). Limnological conditions during this new state are characterized by clearer water with abundant light available for algal growth associated with the rapid development of a benthic biofilm. In addition to this change in habitat availability, there were also abrupt shifts in nutrient balance. Increases in *δ*^13^C_org_ indicate productivity-driven ^13^C-enrichment of DIC, and geochemical and pigment data suggest higher N demand relative to supply. These conditions have persisted to the present at WAP 12, whereas WAP 20 and WAP 21 underwent additional limnological changes beginning in the mid-1970s. Rapid changes in nutrient dynamics and the development of new habitat supporting new algal community composition that persisted for decades in all three ponds are characteristics of a limnological regime shift, which we associate with climate warming at the end of the LIA.

Regime shifts in response to late 19th and early 20th century post-LIA warming have been identified at other Arctic lakes, based on paleolimnological studies, and have mainly identified shifts in aquatic habitat (e.g., Sovari et al. 2002; Rühland et al. 2003; Rühland and Smol 2005; Smol et al. 2005). Common to all of these studies is a shift in the dominant diatom taxa from benthic *Fragilaria* species to more diverse communities in response to post-LIA warming and subsequent reduction in seasonal ice cover. Other studies have suggested that, during the LIA, extensive ice cover resulted in only a narrow moat of shallow open water forming during the summer which allowed *Fragilaria* species to dominate (e.g., Smol et al. 2005; Smol and Douglas 2007). Decreasing ice cover post-LIA resulted in changes to diatom community composition with moss epiphytic taxa dominant in new littoral habitat (Douglas et al. 1994; Antoniades et al., 2005; Keatley et al. 2006) and *Cyclotella* species dominant in deeper lakes with the development of planktonic habitat (Sovari et al. 2002; Rühland et al. 2003; Rühland and Smol 2005). We also find that small epipsammic *Fragilaria* species are outcompeted by taxa commonly found in benthic mats at the end of the 19th century, likely due to decreased ice cover and creation of new available habitat. However, our results also document that this limnological regime shift was accompanied by increased aquatic productivity and low N availability. Increased aquatic productivity and nutrient demand likely led to N-limited conditions and stimulated the growth of benthic biofilms with abundant cyanobacteria, which have been found to dominate under these conditions (Vreca and Muri 2010; White 2011).

An important exception to the widespread evidence of late 19th and early 20th century warming in subarctic and Arctic lakes and ponds is the recent study by Rühland et al. (2013), which used stratigraphic changes in diatom assemblages to suggest that the southern HBL region has undergone post-LIA limnological regime shifts associated with warming only since the 1990s. Additional work by Rühland et al. (2014) provided further support of recent diatom assemblage changes. We suggest that the earlier initiation of the effects of 20th century warming on limnological conditions reported here compared to that reported by Rühland et al. (2013) may be due to the shallow and thus more climatically sensitive nature of northwestern HBL ponds compared to the deep and more resilient southern HBL lakes. As relayed by Rühland et al. (2013), observation that these resilient lakes are now also showing limnological consequences of warming indicates that “climate of the HBL has passed a tipping point” (p. 1).

### Limnological regime shift caused by Lesser Snow Goose population expansion

The LSG-disturbed ponds WAP 20 and WAP 21 both display a third phase in their stratigraphic records beginning in the mid-1970s, which persists to the present but is absent from the undisturbed pond WAP 12. Based on the paleolimnological and water chemistry data, this phase is characterized by increased N availability, but with rapid uptake, increased C demand, and increased productivity. Because these rapid changes in limnological conditions only occurred at WAP 20 and WAP 21 ponds, this suggests a site-specific trigger rather than a regional environmental change. This site-specific trigger led to long-lasting changes in nutrient dynamics and community composition of algae and can therefore be considered a regime shift. The timing of this limnological regime shift corresponds well with the documented expansion of the LSG population (e.g., Batt et al. 1997; Jefferies et al. 2006; Fig.2E), which likely would have provided the source of additional nutrient loading to the disturbed ponds through fecal deposition or indirectly through the alteration of catchment vegetation and habitat (e.g., Batt et al. 1997; Handa et al. 2002; Jefferies et al. 2004; Abraham et al. 2005a).

Based on the patterns in *δ*^13^C_org_ in the sediment records, WAP 21 appears to have been disturbed longer, or more intensely, by the LSG population than WAP 20. At WAP 21, *δ*^13^C_org_ began to decline soon after the LSG population expansion began. We attribute this trend to declining *δ*^13^C_DIC_ caused by chemically enhanced CO_2_ invasion, which occurs under conditions of high productivity, high C demand, and high pH (Herczeg and Fairbanks 1987; Takahashi et al. 1990; Bade et al. 2004). Indeed, while Herczeg and Fairbanks (1987) described this process in their study of the present-day carbon balance of Mohonk Lake, NY, they speculated that negative excursions in the carbon isotope composition of lake sediments may develop from increasing aquatic productivity when chemically enhanced CO_2_ invasion occurs to meet C demand. Conversely, the *δ*^13^C_org_ record at WAP 20 increased after the LSG population began to expand and stabilized at ∽1998, suggesting that chemically enhanced CO_2_ invasion is a more recent and less intense phenomenon at this pond.

Limnological regime shifts in response to nutrient enrichment have been identified in the Arctic (Douglas and Smol 2000; Douglas et al. 2004; Michelutti et al. 2007; Hadley et al. 2010). Many of these studies document nutrient enrichment as a consequence of anthropogenic inputs (e.g., increased sewage inputs from local communities and Thule whaling activities), or other factors in the catchment (Medeiros et al. 2014), rather than directly from activities of wildlife, as inferred from LSG disturbance in our study. While other Arctic paleolimnological and contemporary limnological studies have attributed increases in productivity to disturbance from waterfowl and seabird populations (Michelutti et al. 2009, 2010; Côté et al. 2010; Keatley et al. 2011; Sun et al. 2013; MacDonald et al. 2014), they have not explicitly identified a regime shift. This is likely because contemporary limnological studies alone tend to not have sufficient temporal perspective to document persistence of a new set of conditions. In addition, analysis of an insufficient suite of variables in both paleolimnological and contemporary limnological studies may hinder recognition of regime shifts in internal nutrient behavior and habitat.

### Implications for aquatic ecosystem monitoring

Based on our analyses, we identify a suite of pond responses (physico-chemical, biological) and key indicators based on contemporary limnological and paleolimnological measurements that most effectively defined limnological regime shifts caused by climate warming and catchment disturbance by the expansion of LSG population. We use this as a basis for identifying effective tools for ongoing monitoring of limnological changes in ponds of WNP and elsewhere in response to these stressors (Table2). In our study, consequences of warming were best identified by paleolimnological indicators, namely increasing organic matter content and flux that reflect increasing aquatic productivity, change in composition of diatom assemblages (i.e., switch in dominance from *Fragilaria pinnata* to *Denticula kuetzingii*) that document a shift from epipsammic to benthic habitat, and the appearance of cyanobacteria pigments that indicate more intense competition for dissolved inorganic N. In contrast, limnological consequences of the LSG population expansion were identified from both contemporary and paleolimnological data. High values of conductivity and mid-summer carbon isotope measurements reveal disturbance by LSG on pond catchments, and specifically influx of dissolved ions and nutrients that had a discernible influence on pond-water carbon balance as inferred from carbon isotope data (*δ*^13^C_DIC_, Δ^13^C_DIC-POM_). Interestingly, paleolimnological indicators, including pigments and *δ*^15^N, clearly reveal greater nitrogen availability due to increased nutrient supply, but this is not readily detected by measurements of TKN concentration in pond water, likely due to rapid nutrient uptake by the benthic biofilm (Eichel et al. 2014; MacDonald et al. 2014). Overall, based on our study, we recommend a combination of contemporary limnological and paleolimnological methods for future aquatic ecosystem monitoring initiatives to track responses of Arctic and subarctic ponds to climate warming and expanding wildlife populations. Also, we recommend that research be conducted on a larger number of ponds within Wapusk National Park, both affected and unaffected by LSG disturbance, to further assess the sensitivity of the metrics we identify here and determine whether the regime shifts we identified in a small number of ponds can be generalized to ponds across the landscape.

**Table 2:** The most sensitive limnological and paleolimnological parameters for identifying regime shifts from climate warming and LSG population expansion based on this study

Stressor	Paleolimnology		Limnology	
	Parameter	Interpretation	Parameter	Interpretation
Climate Warming	Organic matter content	• Increase reflects increasing productivity	n/a	n/a
	Diatoms	• Switch in dominance from episammic to benthic mat-dwelling taxa (e.g., Fragilaria pinnata to Denticula kuetzingii) indicates habitat shift		
	Pigments	• Appearance of cyanobacteria pigments (e.g., aphanizophyll) suggests N-limited conditions		
Lesser Snow Goose Expansion	*δ*^15^N	• Increase from 0‰ suggests increased N availability and increased productivity	*δδ*^13^c_dic_	• Mid-summer decline suggests chemically-enhanced CO_2_ invasion due to high productivity and high C demand
	Pigments	• Decrease in cyanobacteria pigment abundance suggests increased N supply	ΔΔ^13^c_dic-pom_	• Decrease in values to below 20‰ suggest high C demand
	*δδ*^13^C_org_	• Decrease suggests chemically-enhanced CO_2_ invasion due to high productivity and high C demand	Conductivity	• High values due to erosional input of dissolved ions
